# Creatine electrolyte supplement improves anaerobic power and strength: a randomized double-blind control study

**DOI:** 10.1186/s12970-019-0291-x

**Published:** 2019-05-24

**Authors:** Erik Hummer, David N. Suprak, Harsh H. Buddhadev, Lorrie Brilla, Jun G. San Juan

**Affiliations:** 1 The University of Tennessee, 1914 Andy Holt Ave, Knoxville, TN 37996 USA; 2 Western Washington University, 516 High St, Bellingham, WA 98225-9067 USA

**Keywords:** Creatine supplementation, Multi-ingredient performance supplement, Strength performance

## Abstract

**Background:**

Creatine supplementation aids the Phosphagen system by increasing the amount of free creatine and phosphocreatine available to replenish adenosine triphosphate. The purpose of this study was to investigate the effects of a creatine and electrolyte formulated multi-ingredient performance supplement (MIPS) on strength and power performance compared to a placebo. Maximal strength along with total concentric work, mean rate of force development (mRFD), mean power, peak power, and peak force for both bench press and back squat were determined at pre-test and post-test separated by 6 weeks of supplementation.

**Methods:**

Twenty-two subjects (6 females, 21 ± 2 yrs., 72.46 ± 11.18 kg, 1.72 ± 0.09 m) performed a one-repetition maximum (1RM) for back squat and bench press. Eighty percent of the subject’s pre-test 1RM was used for a maximal repetition test to assess performance variables. Testing was separated by 6 weeks of supplementation of a MIPS dose per day in a double-blind fashion for comparison. A two-way mixed analysis of covariance (ANCOVA) was applied with an alpha level of 0.05.

**Results:**

For their back squat 1RM, the MIPS group displayed significant increase of 13.4% (95% CI: 2.77, 23.8%) while placebo displayed a decrease of − 0.2% (95% CI: − 1.46, 2.87%) (*p* = 0.047, *η*_*p*_^*2*^ = 0.201). The MIPS displayed a significant increase of 5.9% (95% CI: 2.5, 10.1%) and placebo displayed a non-significant increase of 0.7% (95% CI: − 3.49, 3.9%) in bench press maximal strength (*p* = 0.033,0.217). The MIPS group displayed a significant increase as well in total concentric work (26.5, 95% CI: 6.07, 46.87%, *p* = 0.008, *η*_*p*_^*2*^ = 0.330) and mean power (17.9, 95% CI: 3.42, 32.46%, *p* = 0.003, *η*_*p*_^*2*^ = 0.402) for the maximal repetition bench press test at 80% of their 1RM.

**Conclusions:**

The MIPS was found to be beneficial to recreationally trained individuals compared to a placebo. The greatest benefits are seen in bench press and back squat maximal strength as well as multiple repetition tests to fatigue during the bench press exercise.

## Background

The body utilizes various energy systems to replenish adenosine triphosphate (ATP) during activity. One such energy system, the phosphagen system, is a fast-acting anaerobic energy system [1, 2]. Creatine phosphate is a naturally occurring molecule in the human body which replenishes ATP during rapid movements through the phosphagen system [3–5]. It has been determined that increasing the storage of free creatine and creatine phosphate can help prolong the usage of the phosphagen system [1, 3, 6, 7]. Supplementation of creatine has been shown to enhance the total storage of creatine phosphate and helps increase anaerobic power and strength performances [3, 6, 8].

Creatine supplementation is used to enhance characteristics of physical performance, most commonly strength and power activities [9–11]. Multiple groups have concluded that creatine supplementation, ranging from 1 week to 12 weeks, can be helpful in enhancing one-repetition maximum strength, total work capacity, increasing lean body mass, and, in some cases, increasing lower extremity power output primarily seen in cycling [9, 12, 13].

Electrolytes such as sodium, potassium, and magnesium are transporters used to aid in the absorption and utilization of creatine by the body [14, 15]. While some studies have combined the use of creatine supplementation with beta alanine and other solutions, there is very little literature on creatine supplementation coupled with electrolytes. Brilla et al. [15] examined the effects of a magnesium-creatine supplementation on body water and quadriceps torque. The group supplemented with Mg-creatine chelate displayed significant increases in their intra-cellular water, as well as quadriceps peak torque. Another study aimed to examine the impact of a supplement that consisted of creatine monohydrate (CM) with glucose, sodium, potassium, and taurine (CM + CHO). College aged male football players were tested for bench press strength, vertical jump height, and 100-yard dash times following an 8-week supplementation. Both interventions were compared to a group that only had the glucose and various electrolytes [16]. The CM + CHO groups showed significant increases in their bench press strength, vertical jump, and 100-yard dash times to the CHO only group, and larger relative increases compared to the CM only group [16]. Other authors have reported that creatine uptake is directly impacted by the presence of sodium, chloride, and calcium [17–20]. Dai el al. found that when both calcium and magnesium were absent from an extracellular fluid, creatine uptake was significantly reduced by 47%. The same authors also found that in a similar fluid, creatine uptake is increased when concentrations of sodium and chloride are increased, even when creatine concentration remains constant [17]. For creatine transportation to occur, the body requires both sodium and chloride ions to transport the creatine molecules. Supplementing creatine with these electrolytes aids with increased uptake and storage of muscular creatine, thus potentially increasing performance.

The purpose of this study was to investigate the effects of a creatine supplement formulated with various electrolytes on upper and lower limb anaerobic power, and strength outcomes for college-aged individuals. We hypothesized that a six-week supplementation period of the multi-ingredient performance supplement (MIPS) would significantly increase predicted one-repetition maximum (1RM) for back squat and bench press. Additionally, we expected an increase in the amount of concentric work, mean power, peak power, mean rate of force development (MRFD), and peak force post MIPS supplementation for back squat and bench press during a maximal repetition test at 80% 1RM load.

## Methods

### Study design

A double blind, randomized, pre- and post-test repeated measures design was implemented to examine the effects of MIPS on upper and lower limb strength and power performance measures in recreationally trained college-aged students. The research protocol was approved by the institutional review board of Western Washington University (Protocol IRB # 16–002). A total of 6 weeks’ supplementation was completed between pre-test and post-test measurements to ensure tissue saturation to elicit possible effects of the intervention [9, 13, 21].

### Subjects

Twenty-two healthy subjects (16 males, 6 females) aged 19–24 years were included in this study. All subjects were regularly strength training for a minimum of 6 months prior to the study, free of any injury that would inhibit training, creatine supplementation-free for at least 1 month prior to participation, and free from any endocrine or kidney disease that would impact their clearance of creatine. Subjects were required to participate in strength training 2–3 days per week to fit our criteria of being recreationally strength trained. Subjects were randomly assigned into two groups, the experimental MIPS group (*n* = 12) and the placebo group (*n* = 10). Subjects were randomly assigned either a 1 or 2 dictating which group they would be allocated too. Randomization of group assignments were done prior to subject recruitment; thus, sequential subjects could be placed into the same group. The MIPS supplement was formulated with the following: 4 g of creatine, 857 mg of phosphorus, 286 mg of magnesium, 171 mg of calcium, 171 mg of potassium, and 114 mg of sodium. The MIPS supplement was provided directly to the principal investigators by the manufacturer for product evaluation and is not yet commercially available during the time of testing. The placebo group received 5599 mg of maltodextrin in a similar package.

### Data collection

Height was recorded using a stadiometer and mass was measured using a calibrated weighing scale (COSMED, Rome, Italy). The barbell trajectories during bench press and back squat movements were collected using Qualisys Track Manager 2.7 (Qualisys Motion Capture Systems, Gothenburg, Sweden) and seven Qualisys Proreflex MCU 240 cameras (Qualisys Motion Capture Systems, Gothenburg, Sweden) at a sampling frequency 240 Hz. Kinetic data for the akimbo countermovement jumps were collected using an imbedded force platform (AMTI, Watertown, MA, USA) collecting at 50 Hz. All testing for back squat and bench press were performed in a squat rack (PR Basic Squat Rack, PR Lifting, Everett, WA, USA).

Subjects began with a five-minute warm up on a cycle ergometer (Star Trac, Orange County, California, USA) choosing their own pace and resistance. Following the cycle ergometer warm up, subjects performed a dynamic warm-up consisting of five repetitions of the following: knee hugs bilaterally, Frankenstein’s bilaterally, walking quadriceps stretch bilaterally, lunge with a torso twist bilaterally, wall slides, shoulder slaps bilaterally, and push-ups. Following the warm up, subjects were tasked with performing three akimbo countermovement jumps while on a force plate. Subjects were shown how to perform the movements and allowed practice trials until they were comfortable.

Subjects then watched a pre-recorded video detailing how to perform the back-squat exercise made by the Western Washington University Strength and Conditioning coach. Subjects were instructed to perform the back squat to 90° of knee flexion and then return to the upright position. The testing for the back squat 1RM followed the National Strength and Conditioning Association guidelines for predicting a 1RM [22]. Subjects were spotted for every set they performed during testing. Subjects estimated their current 1RM for the back squat based on their previous strength training program. This estimation was used for the loading during the 1RM test, with a new 1RM being calculated using the O’Connor 1RM estimation equation [23]. Subjects were given four-minute rest periods between their sets. On the final set of the 1RM test, subjects were told to go until failure or until they decided they could not continue. Subjects were given a four-minute rest period before moving on to the bench press 1RM test. Subjects performed the same protocol for the bench press 1RM test as the previously performed back squat 1RM test. The bench press range of motion was defined as fully extended elbows to the point at which the barbell made contact with the chest. Participants were given an instructional video detailing the bench press exercise by the same Western Washington Universities Strength and Conditioning coach.

Following 1RM bench press testing, 80% of the subject’s 1RM bench press was determined, the load was adjusted on the barbell, and placed inside the motion capture system capture area. One retro-reflective marker was placed on the end of the barbell to track the trajectory of the barbell. Subjects were instructed to perform as many repetitions as possible and to perform them as forcefully as possible. Data collection commenced when the subject removed the weight from the rack prior to initiating the first repetition. Subjects performed as many bench press repetitions as possible and returned the barbell to its resting position. Subjects were given an eight-minute rest before beginning the back squat maximal repetition test. The back-squat load was adjusted to reflect 80% of their predicted back squat 1RM from the previous test. As with the bench press testing, subjects were instructed to perform as many repetitions as possible and to perform them as forcefully as possible. When testing concluded, subjects received a briefing of the supplementation protocol and their obligations. Subjects were asked to take a single dose per day, maintain their typical and current training program, and to return in 6 weeks for their post-testing data collection.

### Supplementation protocol

Supplementation occurred for a total of 6 weeks with subjects taking one dose per day of treatment or placebo according to group with 16 oz. of water, accompanied with a meal following a workout. No loading dose phase was conducted as lower maintenance does over a period of 6 weeks would yield equivalent results of increasing creatine levels in muscular tissues [7, 24]. Subjects were instructed to maintain their current and typical training program. Following the 6 weeks of supplementation, subjects returned to perform post-testing measurements. Post-testing procedures were kept similar except for the load utilized for the maximal repetition test, which used 80% of pre-testing 1RM. Subjects were removed from participation if they reported missing greater than 3 days of supplementation.

### Data analysis

The trajectories of the barbell during bench press and back-squat tests were processed using a 4th order low pass Butterworth filter at a cutoff frequency of 6 Hz and exported into a text file for further analysis. The text files were analyzed in a custom LabVIEW (National Instruments, Austin, TX, USA) program that identified the concentric phases of completed repetitions before computing the total work, mean power, peak power, mean rate of force development, and peak force. Once the concentric phase of movement was determined, the LabVIEW program took the sum of work (force * displacement) for each repetition to obtain total concentric work. Mean power was obtained by averaging the product of force * velocity at each time point during the repetition and set. Similarly, peak power was obtained from the maximal product of force and velocity during each repetition, with the greatest value being used for data analysis. mFRD was determined from the slope of the force-time curve to the peak force during the concentric phase.

### Statistical analysis

Data were analyzed between the experimental MIPS supplement group and the placebo group at pre-test and post-test. The groups were initially compared at pre-test using a one-way analysis of variance (ANOVA) with an alpha of 0.05 to ensure there was no significant effect of group, and therefore no significant randomization effect. Preliminary statistical analysis revealed that gender had a significant effect on the dependent variables. To control for the confounding effects of gender, it was used a covariate for all subsequent analysis, A two-way mixed analysis of covariance (ANCOVA) with an alpha level of 0.05 compared group and time on the following: back squat 1RM, bench press 1RM, and concentric work, mean power, peak power, mRFD, and peak force during a maximal repetition test done at 80% 1RM for back squat and bench press. Simple effect analyses were run for significant interactions and pairwise t-tests were run for present main effects. Before the ANCOVA was run, all data was tested for normality (Shapiro-Wilks), homogeneity of variance (Levene’s test of equality of error variance), and sphericity (Mauchly’s). When interactions were present, post-hoc analysis was run using pair-wise t-tests. Effect sizes were reported as partial eta squared (η_p_^2^) and follow the guidelines outlined in Vincent [25]. A η_p_^2^ of greater than 0.15 was large, between 0.06 and 0.15 was medium, and lower than 0.06 was small. All data were analyzed using SPSS (version 25).

## Results

A total of 22 participants completed both pre- and post-testing sessions separated by 6 weeks supplementation. Age, sex, height, and mass were all recorded at pre-test and post-test to describe the samples for both the placebo and MIPS groups (Table 1). The assumptions of normality and homogeneity of variances remained unviolated for the dependent variables. Greenhouse-Geisser corrections were taken into account when the assumption of sphericity was violated.

**Table 1:** Subject demographic characteristics pre- and post- test

	Placebo		MIPS	
	Pre-test	Post-test	Pre-test	Post-test
Sex	8 M, 2 F		8 M, 4 F	
Age (yr)	20.70 ± 1.49	20.7 ± 1.49	21.92 ± 2.71	21.92 ± 2.71
Height (m)	1.74 ± 0.11	1.74 ± 0.11	1.70 ± 0.08	1.70 ± 0.08
Mass (kg)	73.10 ± 13.56	72.97 ± 14.26	71.82 ± 8.84	71.55 ± 6.48

Values are displayed as mean ± one SD

The initial one-way ANOVA displayed no significant differences between the placebo and MIPS groups at pre-test. Back squat 1RM (*p* = 0.54), bench press 1RM (*p* = 0.28), bench press concentric work (*p* = 0.72), mRFD (*p* = 0.08), mean power (*p* = 0.81), peak power (*p* = 0.50), peak force (*p* = 0.95) and back squat concentric work (*p* = 0.67), mRFD (*p* = 0.08), mean power (*p* = 0.72), peak power (*p* = 0.68), peak force (*p* = 0.73) where not significantly different at pre-test.

**Fig. 1:**
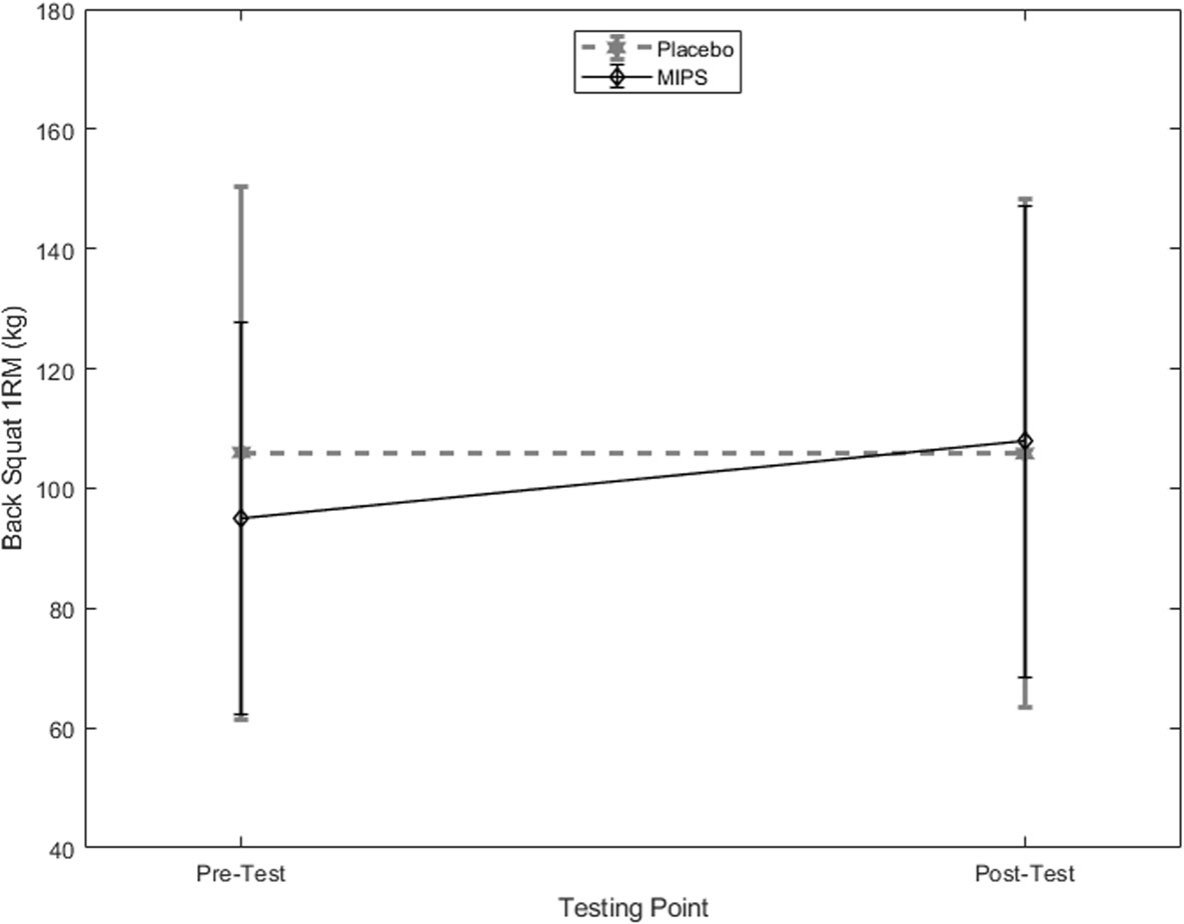
Back Squat 1RM (kg) for the placebo and MIPS groups, pre- and post-supplementation (mean ± SD)

**Fig. 2:**
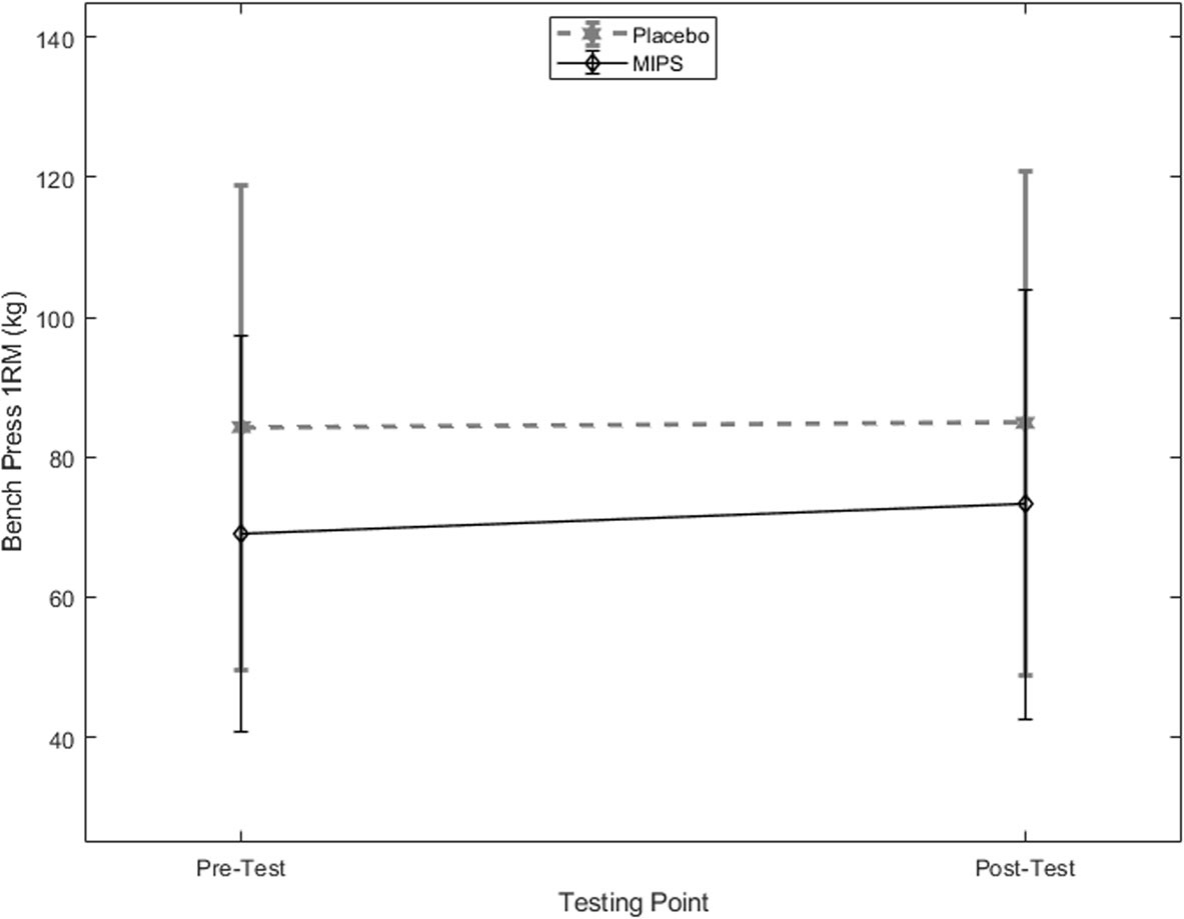
Bench Press 1RM (kg) for the placebo and MIPS groups, pre- and post-supplementation (mean ± SD)

There was a significant interaction between time and group for back squat 1RM (*p* = 0.047, *η*_*p*_^*2*^ = 0.201) (Fig. 1). From pre- to post-testing, the MIPS group increased their back squat 1RM significantly by 13.4% (95% CI: 2.77, 23.8%) and the placebo group displayed a slight decrease of − 0.2% (95% CI: − 1.46, 2.87%). There was also a significant interaction between time and group for the bench press 1RM (*p =* 0.033, *η*_*p*_^*2*^ = 0.217) (Fig. 2). The MIPS group displayed a significant increase of 5.9% (95% CI: 2.5, 10.1%) while the placebo group increased by 0.7% (95% CI: − 3.49, 3.9%).

There were no significant interactions or main effects of time or group during the back squat maximal repetition test for any of the following: sum of concentric work (*p* = 0.229, 0.700, and 0.855, respectively), mRFD (*p* = 0.630, 0.653, and 0.215, respectively), mean power (*p* = 0.405, 0.884, and 0.897, respectively), peak power (*p* = 0.219, 0.064, 0.975, respectively), or peak force (*p* = 0.349, 0.097, 0.998, respectively).

There was a significant interaction between time and group for sum of concentric work during the bench press repetition test (*p* = 0.008, *η*_*p*_^*2*^ = 0.330). The MIPS group displayed an increased sum of concentric work of 26% (95% CI: 6.07, 46.87%) compared to a slight decrease of − 3.4% (95% CI: − 15.36, 8.63%) for the placebo group (Table 2). There was no significant interaction between time and group for mRFD (*p* = 0.101, *η*_*p*_^*2*^ = 0.142). There was a significant interaction between time and group for mean power (*p* = 0.003, *η*_*p*_^*2*^ = 0.402). The MIPS displayed an increased mean power of 17.9% (95% CI: 3.42, 32.46%) while the placebo displayed a decreased mean power of − 3.4% (95% CI: − 8.75, 2.09%) (Table 2). There was no significant interaction or main effect of time or group for peak force during the maximal bench press test (*p* = 0.355, 0.979, 0.955, respectively).

**Table 2:** Bench press maximal repetition test results pre- and post-test

	Placebo		MIPS	
	Pre-Test	Post-Test	Pre-Test	Post-Test
Sum of Concentric Work (J) ^*^	1990.6 ± 864.7	1923.6 ± 703.8	1853.9 ± 829.8	2344.7 ± 1121.2
Mean Rate of Force Development (N/s) ^*^	7293.9 ± 6240.7	5385.4 ± 5034.0	3620.4 ± 2358.7	4432.0 ± 3152.2
Mean Power (Watts) *	197.9 ± 113.6	191.3 ± 110.4	169.7 ± 73.6	200.1 ± 83.0
Peak Power (Watts)	1204.7 ± 604.0	1295.8 ± 629.1	1134.8 ± 679.6	1275.5 ± 579.8
Peak Force (N)	2524.0 ± 1141.8	2789.0 ± 1363.9	2564.9 ± 1450.8	2507.6 ± 1074.7

Values are mean ± one SD

^*^significant interaction of group and time (*p* < 0.05)

## Discussion

The purpose of the study was to examine the effects of a creatine electrolyte MIPS on strength and power in recreational strength trained individuals. We compared two supplementation groups, one was MIPS formulated with creatine and electrolytes and the other group had placebo formulated with only maltodextrin, before and after 6 weeks of supplementation. Measurements of maximal strength (1RM) as well as other biomechanical performance variables during the bench press and back squat. Our primary hypothesis was supported, the MIPS group displayed a significant increase to their back squat and bench press 1RM compared to the placebo group.

The MIPS group increased the maximal strength for both the back squat and bench press exercises following 6 weeks of supplementation. In agreement with this finding, Hoffman et al. [26] found an increase in back squat 1RM of approximately 15.6%, for groups supplemented with creatine monohydrate for 10 weeks using 10.5 g^.^d^− 1^. Pearson et al. [27] also found a significant increase in back squat 1RM following 10 weeks of creatine monohydrate supplementation. Subjects were given 5 g^.^d^− 1^ for the supplementation period and experienced a significant increase of 11.5% in their back squat 1RM [27]. Another study tested maximal back squat strength in female soccer players at five and 13 weeks of creatine monohydrate supplementation [2]. Supplementation consisted of a 15 g^.^d^− 1^ for one-week loading phase followed by a four-week maintenance phase of 5 g^.^d^− 1^. The soccer players displayed significant 23.6% increases in their 1RM back squat following 13 weeks of creatine supplementation [2]. These results are in line with the current data, which showed an increase of 13.4% in back squat strength following only a six-week MIPS intervention. The literature and current study seem to agree that creatine supplementation lasting at least 5 weeks can increase back squat maximal strength across various populations ranging from recreational to competitive athletes. Benefits from the MIPS were also very similar to those using creatine monohydrate at relatively similar doses (4–5 g^.^d^− 1^).

The current study displayed that subjects in the MIPS group significantly increased their bench press 1RM by 5.9% following 6 weeks of supplementation. Our results are in agreement with other studies that displayed significantly increased the upper body maximal strength in a range of active people following creatine supplementation [9, 22, 26, 28, 29]. Hoffman et al. [26] found an increase in bench press 1RM of 13%, for groups supplemented with creatine monohydrate for 10 weeks using 10.5 g^.^d^− 1^. This increase amounted to a two-and-a-half-fold increase compared to their placebo counterparts. Another study using 28 days of creatine supplementation found a 6% increase in bench press 1RM when compared to their baseline values [9]. Pearson et al. [27] used a 5 g^.^d^− 1^ creatine supplementation over 10 weeks and found that bench press maximal strength significantly increased by 3.4%. A recent study involving recreational bodybuilders used 5 g^.^d^− 1^ doses of creatine monohydrate and observed an increase in bench press 1RM over a four-week period by 7.5% [30].

Our secondary hypothesis was partially supported, as the MIPS group significantly increased their total concentric work and mean power during the bench press maximal repetition test. Very few studies using creatine supplementation have been done using the performance variables in the current study performing repetitions to failure under certain loading conditions [13, 21, 31]. A 28-day supplementation study using male powerlifters examined a carbohydrate-protein creatine supplements effect on maximal repetition tests across five subsequent sets of bench press [21]. Following supplementation, the groups supplementing with creatine had an increased number of repetitions in sets one, four, and five [21]. This finding could primarily be because of creatine supplementation increasing PCr, since the repetition test occurring at 80% 1RM is heavily dependent on the phosphagen system [13]. The phosphagen system is typically only predominant for 10–15 s and plays a large role in repeated bout exercises. An increase in the amount of creatine available can prolong the usage of the phosphagen system, thus increasing performance over the latter repetitions. The current study only used a single set at 80% of subject’s determined 1RM to closely examine the effect of MIPS supplementation on maximal repetitions to failure. A more recent study examined a similar protocol of performing repetitions to failure at 80% 1RM [13]. Following 30 days of supplementation, both groups supplemented with polyethylene glycosylated creatine (1.25 and 2.50 g^.^d^− 1^ doses) increased their repetitions to failure by 25.7 and 21.9%, respectively [13]. This result suggests that maybe a larger dose of creatine does not always indicate a greater benefit. The significant increase of 26.5% in the current study appears to be in line with current knowledge of creatine increasing the work capacity until failure while performing the bench press exercise at 80% 1RM.

The current data indicated a non-significant, but large effects size (*η*_*p*_^*2*^ *=* 0.16*)* increase in mRFD and a significant increase of mean power during the bench press maximal repetition test for the MIPS group. A maximal repetition test at this specified load is heavily dependent on the phosphagen system [21]. While no research was found on mRFD during the bench press, an increase of mRFD shows a possibility of decreased rate of fatigue. Subjects that displayed an increased mRFD were achieving their peak force at a much faster rate throughout their repetition test. Mean RFD has two major components that could be influenced due to fatigue: velocity and force [32]. With an increase in mRFD, it is possible that MIPS participants were able to maintain either their peak force applied to the barbell, as well as the rate at which the force was achieved. Both considerations are heavily influenced with fatigue, as fatigue takes effect, both the magnitude of force and speed of the movement should reduce. The results could indicate an increased performance during repetitions to failure, with the fatigue being minimized during the post intervention testing.

Most available information on creatine supplementation primarily involves an isolated creatine supplement. This current study combined both creatine and various electrolytes that potentially increase the absorption of creatine, increase transport into the muscle, and increase performance. Therefore, our results may not be directly relatable to a creatine only supplement, due to the addition of electrolytes in the current MIPS. However, Brilla et al. [15] supplemented participants using a magnesium-creatine supplement and observed increases in quadriceps peak torques along with increased intra-cellular water content. This finding could be potentially related to our current study displaying an increased back squat 1RM. The back-squat exercise is a quadriceps dominant movement, requiring greater knee extensor torques to overcome a greater amount of weight. Crisafulli et al. [33] examined a similar electrolyte creatine supplement on repeated sprints during cycling. Cycling performance (peak and average power output) during sprint cycling were increased following 6 weeks of an electrolyte creatine supplement. While not directly comparable, the current information merits further research on creatine supplemented that include electrolytes.

### Limitations

There were some limiting factors to the current study that could have influenced the results. The maximal repetition strength test was conducted using as many repetitions as possible at 80% of their predicted 1RM. Subjects were instructed to refrain from strenuous exercise prior to testing, but this was not controlled. There was also no control used for back squat depth for subjects to maintain. They were instructed to squat until 90° knee flexion angle was elicited, but no objective protocol to control for depth was utilized. The maximal repetition test for the back squat occurred last in the data collection, and therefore could be affected by accumulative fatigue from the testing procedures. Additionally, the supplement was not tested by an individual third party laboratory to confirm the potency of the supplement prior to the start of the study. Greater adherence to the diet logs provided would have examined the potential impact of diet during the supplementation period and ensure a change in diet did not impact the results. One other potential limitation could be the use of maintenance only doses of supplementation. However, this limitation is minimal as other studies have found that maintenance doses over longer periods of time have the same effect of creatine saturation in muscular tissue [24]. Finally, comparing the MIPS group to a creatine only group would have added to the comparison to examine the effects of electrolytes directly. Our results are in line with previous creatine versus placebo studies and merit more follow up with a direct comparison to a creatine only supplement.

Future research could focus on expanding to more resistance training exercises using similar repetitions to failure that rely greatly upon the phosphagen system. Creatine supplementation has been noted to have the greatest effect on repeated bout exercises, with multiple repetitions with moderated rest [13, 21, 31, 34]. Comparing the MIPS to a creatine monohydrate supplement would also be a next step to compare if the electrolytes elicit a significant effect compared to a creatine monohydrate supplement.

## Conclusion

Six weeks of MIPS supplementation can be beneficial for increasing multiple facets of athletic performance in recreationally trained individuals. The MIPS increased both back squat and bench press maximal strength. Subsequently, the MIPS also displayed increases of concentric work and average mean power while performing a maximal repetition test of bench press at 80% 1RM load. This MIPS consisting of creatine and electrolytes could be beneficial for people wishing to increase their performance. Additional studies are needed to compare the MIPS to a creatine only supplement to examine if the performance increase is directly related to the electrolytes in the MIPS.

## Data Availability

All data pertaining to the conclusions of the study are found within the article. The corresponding data set used is available under reasonable requests.
